# Medical Items

**Published:** 1912-06

**Authors:** 


### MEDICAL ITEMS

### THE WEAKLINGS AMONG CHILDREN.

Boy weaklings particularly have a hard row to hoe. Boys are little savages. The weak ones among them are always subjected to trials their stronger companions never suffer. It is only fair to a child, when it shows evidences of lowered physical endurance, to aid it in every possible way. Frequently the processes of metabolism may be stimulated by a well chosen reconstructive, putting the youngster well on the road to full vigor and helping him hold his own in the boy's world, a world where only physical prowess rules.

In these youthful cases of physical incapacity, the benefit following the long continued administration of NUTROMUL (Brown's Cotton Seed Oil Emulsion), is not only surprising but also truly gratifying. The tissues take up and convert to good use the nutritious properties of the cotton seed oil in NUTROMUL, and it is not long before the child takes on weight, and shows a marked improvement in general health. The addition of the hypophosphites of lime, soda, and manganese increases the potency of the cotton seed oil in NUTROMUL. Another feature is NUTROMUL'S palatability, and the willingness with which children take it over long periods of time. A sample bottle may be had by addressing a postal card to Nottoc Laboratory, Atlanta, Ga.

---

### THE LOGICAL AID IN NERVOUS BREAK-DOWN.

That agent which will tranquilize a hightly wrought up nervous system and aid it in regaining its normal functions, is the one whose employment in nervous break-downs is logically indicated. Such a product is PASADYNE (Daniel's Concentrated Tincture of Passiflora Incarnata), and its therapeutic activity in just such conditions as the one being discussed, has earned for it a leading place among the remedial agents used in nervous disorders. It serves the two-fold purpose of reducing nervous strain and restoring the normal tone of the weakened nervous tissues. A sample bottle will be furnished if application be made to the Laboratory of John B. Daniel, Atlanta, Ga.

---

### THE INSOMNIA OF ALCOHOLISM.

Of all the insomnias, the most difficult to control is that of acute alcoholism. Not only is there wakefulness during the period of convalescence from the restless tossing to and fro, remorse and gloomy anticipations of the future. In these cases the more recently discovered hypnotics do not reach the bottom of the trouble. Bromidia has a profound influence on the entire nervous system, and exercises its sedative effect thereon before the actual hypnotic result is prolonged. The ultimate result is curative for the effect on the brain and cord does not immediately wear off. Furthermore, far from causing anorexia, as similar agents are prone to do, Bromidia actually increases the appetite, a very valuable help in these cases where it is important to build up the system as soon as possible. In all alcholic cases, the use of Bromidia is greatly to be preferred to the hypodermic injection of morphine, with its inevitable result of locking up the secretions, and its frequently disastrous action on the stomach. If opium must be used a dose of Papine will accomplish what is desired without any of the bad effects of morphine.

---

WE ARE FORTUNATE!

HAVING secured the Sole Agency in Atlanta for the complete line of

SHERMAN'S BACTERINS,

We can give you prompt and efficient service.

This list comprises no less than 40 varieties and combinations and there is only one price, 25 cents a dose (regardless of size.)

*The Bacterial Therapist*, a live monthly journal of Vaccine Therapy, will be sent to you for one year on request—no money, no obligation.

PERRYMAN & CO.

10 N. Broad St. Atlanta, Ga. Phone Main 2010

